# Market *versus* Residence Principle: Experimental Evidence on the Effects of a Financial Transaction Tax

**DOI:** 10.1111/ecoj.12339

**Published:** 2017-10-24

**Authors:** Jürgen Huber, Michael Kirchler, Daniel Kleinlercher, Matthias Sutter

**Affiliations:** ^1^ University of Innsbruck; ^2^ University of Innsbruck, University of Gothenburg; ^3^ University of Cologne, University of Innsbruck

## Abstract

The effects of a financial transaction tax (FTT) are scientifically disputed, as seemingly small details of its implementation may matter a lot. In this article, we provide experimental evidence on the different effects of an FTT, depending on whether it is implemented as a tax on markets, on residents, or a combination of both. We find that a tax on markets has negative effects on volatility and trading volume, whereas a tax on residents shows none of these undesired effects. Additionally, we observe that individual risk attitude is not related to traders’ reaction to the different forms of an FTT.

Few other issues stir emotions as easily as ‘taxes’. This also holds for a financial transaction tax (FTT) – dubbed ‘Robin Hood tax’ by its supporters but fiercely contested by others as supposedly threatening to cripple the financial sector. Especially since 11 member countries of the European Union have been considering implementing an FTT by 2015, the discussion about the effects of an FTT has gained momentum.^1^ Such a tax is politically highly controversial, because it has rarely been implemented in practice. Hence, evidence on its likely effects is still very limited.

The academic debate has missed some important institutional details so far, for which reason it cannot provide unambiguous evidence as a basis for the political debate. In particular, the academic literature on an FTT has practically ignored the exact taxation scenarios, i.e. whether such a tax is implemented on all trades in a given market – which we call the ‘market principle’ – or on all trades by residents in a particular jurisdiction – which we call the ‘residence principle’.

In this article, we explore the consequences of applying these different taxation principles on market outcomes such as trading volume, tax revenues, volatility and price efficiency, as well as individual trading behaviour. We do so in a controlled laboratory experiment, using the laboratory as a ‘wind‐tunnel’ environment to test how the market principle, the residence principle, or a combination of both, influence the variables under consideration In particular, experimental subjects can trade assets for money in two independent jurisdictions, each with one financial market. We implement either a tax on residents, a market tax, a combination of a market tax and a tax on residents within the same jurisdiction, or a tax on residents for one jurisdiction and a market tax on the other. What we cannot explore with our setting, however, is the overall allocative efficiency as well as the level of risk‐sharing.

We find that applying the residence principle – meaning that all trades of residents of one jurisdiction are taxed, irrespective of whether they trade on their home market or on the foreign market (an approach similarly discussed in the European Union) – has no significant effects on trading volume or volatility. Thus, it causes practically no distortions on the markets and tax revenues are substantial. When the market principle is applied – i.e. all transactions in one market are taxed, while the other market is not taxed – we observe a significant shift in trading volume: about three quarters of trading in the taxed market shift to the untaxed alternative. With liquidity in the taxed market evaporating, volatility increases significantly, while it drops in the untaxed market where liquidity increases.

The combination of both principles in one jurisdiction leads to a significant drop in trading volume in the jurisdiction implementing both market and residence principle, and an increase in the other one. By contrast, volatility increases in the jurisdiction applying the market and residence principle and drops in the one without any tax burden. However, the overall market distortion is weaker compared to the sole implementation of the market principle but clearly higher compared to jurisdictions implementing only the residence principle.

In the last taxation scenario where one jurisdiction applies the market principle and the other one the residence principle, trade shifts from the jurisdiction with a market tax to the one where only the residents are taxed, causing market distortions within the jurisdiction that applies the market principle.

In addition to disentangling the effects of a market or residence principle, another contribution of our article is to show how individual traders with different attitudes towards risk are influenced by the introduction of an FTT. We find that traders with high risk tolerance trade significantly more than strongly risk‐averse traders. However, risk attitude is irrelevant for a subject's reaction to FTTs: risk seeking and risk averse traders are equally affected by the introduction of an FTT. We consider these insights into an individual level as important for a deeper understanding of how an FTT affects market outcomes. Remarkably, this microfoundation has been absent in previous experimental work on an FTT.

The remainder of the article is structured as follows: In Section 1, the most closely related literature is briefly discussed. In Section 2 the experimental design is introduced. In Section 3 we present results on the aggregate market level, while in Section 4, we look at individual level data. Finally, Section 5 concludes.

## 1 Related Literature

In 1936, John Maynard Keynes first advocated the introduction of an FTT on stock markets as the best way to mitigate the predominance of destabilising short‐term speculation over stabilising long‐term investment (Keynes, 1936). After the fall of the Bretton‐Woods system, a similar line of argument was adopted by James Tobin, when he called for the introduction of an FTT on foreign exchange markets to curb excessive speculation (Tobin, 1978).^2^ Notably, neither Keynes nor Tobin supported their proposals with empirical or analytical research.

This fact did not change until the late 1980s when scientific research on the impact of an FTT of the market principle‐type gained momentum (Stiglitz, 1989; Summers and Summers, 1989; Schwert and Seguin, 1993). Since then there is broad scientific consensus on the negative effects of an FTT of the market principle‐type on trading volume. Other important issues, namely the impact of an FTT on volatility and price efficiency, are still controversially debated, with strong academic supporters for both sides. In one of the earliest empirical contributions Umlauf (1993) reports an increase of price volatility after Sweden introduced a round trip tax on equity transactions in 1984.^3^ Aliber *et al*. (2003) empirically investigate the impact of the size of transaction costs on volatility and show that higher transaction costs are associated with higher volatility. More recently, contributions by Ehrenstein (2002), Westerhoff (2003) and Ehrenstein *et al*. (2005) provide evidence that an FTT drives chartists from the taxed market and hence stabilises prices.^4^ Turning to the effects of an FTT on price efficiency, Cipriani and Guarino (2008) and Bloomfield *et al*. (2009) study the effects of an FTT in an experimental financial market. The former find an increase in informational efficiency but hardly any effects on market volatility. The latter investigate the effects of an FTT on price efficiency through informational cascades. They report no effects on price efficiency in their experiments. In contrast, theoretical work by Subrahmanyam (1998) and Dow and Rahi (2000) concludes that an FTT would decreases price efficiency. To sum up, there is no agreement on the consequences of an FTT of the market principle‐type on price volatility and price efficiency.

A limitation of many of the papers mentioned so far is that they consider only one market. While such papers are useful to understand how a tax affects aggregate market outcomes, they are obviously limited to cases where an FTT would cover all existing markets – a scenario that fails to match the current real‐world situation. For this reason, recent work has started to examine a setting with two or more markets, because that allows for the coexistence of taxed and untaxed markets.

In agent‐based simulations with two markets, Westerhoff and Dieci (2006) and Mannaro *et al*. (2008) analyse the effects of an FTT either implemented as encompassing or as a unilateral tax, i.e. where a tax haven exists. Westerhoff and Dieci (2006) use agents applying technical and fundamental analysis for trading on two different markets. When an FTT is levied on one market they show that volatility decreases in the taxed market and increases in the untaxed one. In contrast, with a different agent‐based modelling approach, Mannaro *et al*. (2008) argue that the higher an FTT, the higher the increase in volatility in the taxed market.

Hanke *et al*. (2010) use laboratory markets to investigate the effects of an FTT. They report that an FTT only imposed on one market increases volatility when the market is small and illiquid but has no impact on volatility when the market is large and liquid. Thus, Hanke *et al*. (2010) stress the crucial interplay of liquidity and volatility when an FTT is imposed.^5^ This important relationship is also addressed by Pellizzari and Westerhoff (2009) and Kirchler *et al*. (2011). Both focus on the market microstructure as an important issue regarding the effects of an FTT. Pellizzari and Westerhoff (2009) show – in the framework of a one‐market agent‐based model, though – that in a dealership market where liquidity is held constant through artificial market makers an FTT has no negative effects on volatility. By contrast, in a taxed double‐auction market volatility rises as soon as liquidity drops. Because of a lower orderbook depth, buy and sell‐orders have a greater price impact which makes prices more volatile. Kirchler *et al*. (2011) tackle this question with laboratory experiments where traders can trade on two simultaneously running financial markets. They conclude that in markets without market makers an unilaterally imposed FTT increases volatility, while in markets with market makers – and therefore constant liquidity – a unilaterally imposed FTT even decreases volatility. Hence, again there is no consensus on the consequences of an FTT of the market principle‐type on price volatility and price efficiency in the academic literature.

So far no paper has explored the effects of an FTT implemented according to a residence principle, market principle or as mixture of market and residence principle, leaving it an open question how the institutional details of an FTT matter for its effects. We fill this gap with this article, concentrating not only on aggregate market outcomes in our analysis but also on how an FTT affects individual trader behaviour.

## 2 Design of the Experiment

The fundamental value of the asset traded (expressed in Taler) is modelled as geometric Brownian motion:

(1) $${\text{FV}}_{k}={\text{FV}}_{k-1}\times e^{\gamma_{k}}.$$

FV_*k*_ denotes the fundamental value in period *k* and $\gamma_{k}$ is a normally distributed random variable with a mean of zero and a standard deviation of 10%. The FV_0_ is set to 40. We draw one fundamental value path randomly (path I) and then create a counterpart by mirroring path I at the unconditional expected value of the FV.^6^ In half of the sessions for each treatment, we use path I, in the other half path II. Furthermore, we introduce a symmetric information structure. In each period each subject receives a private signal on the fundamental value of the asset. This signal is calculated as the current FV plus a noise term with a mean of zero and a standard deviation of 5%. Estimation errors cancel out across subjects to make sure that each market has an unbiased estimation of the FV.^7^

The treatments are designed to test the effects of a financial transaction tax (FTT), either implemented as a tax on each transaction conducted in a given market (market principle), or a tax on each transaction by a person hailing from a given jurisdiction (residence principle), or a combination of both.

Subjects can trade units of one asset on two different markets (denoted LEFT and RIGHT). Subjects are assigned to one market as their home market, i.e. half of the subjects are residents of market LEFT (home market LEFT) and the other half are residents of market RIGHT (home market RIGHT). This enables us to tax transactions on a particular market or the residents of a given market (or jurisdiction), respectively, within various taxation scenarios.

As a preliminary before presenting design details, we provide the following definitions: A session consists of two markets (LEFT and RIGHT) where 10 subjects can trade simultaneously for a sequence of eight periods. These are divided into two phases of four consecutive trading periods where a certain taxation scenario is levied. A taxation scenario (treatment) specifies how an FTT of 0.1% is collected, i.e. either as a tax on transactions in a given market, as a tax on residents of a given market (jurisdiction), or a combination of both.

Each session is populated by 10 subjects and has eight periods of four minutes trading.^8^ Subjects trade units of the asset on two continuous double auction markets simultaneously. Both markets (LEFT and RIGHT) are displayed on the trading screen at the same time. It is possible to buy assets on the right market and to sell them on the left market, or *vice versa*, as it is possible to buy or sell assets on the same market.

### 2.1 Treatments

We implement four treatments which only differ with respect to the taxation scenarios.

#### 2.1.1 Treatment M: market principle

This taxation scenario follows the proposal of Tobin (1978) to introduce an FTT on financial markets as a market tax. This means that each trade on the taxed market is taxed, irrespective of the residence of the involved traders. For the sake of simplicity we only tax market LEFT, while market RIGHT serves as tax haven.

#### 2.1.2 Treatment R: residence principle

This taxation scenario follows the idea of imposing an FTT on residents of a given market (jurisdiction). Every market participant who is resident in the jurisdiction that levies an FTT is taxed for all his trading activities, no matter whether these are conducted on the domestic or a foreign market. In particular, subjects who are residents of the left market (home market LEFT) are taxed for each trade they make, no matter whether it happens on the left or right market. Subjects with residence on the right market (home market RIGHT) can trade on the left and right market without being taxed.

#### 2.1.3 Treatment MR_SAME_: market and residence principle on the same market

Treatment MR_SAME_ is a combination of treatments M and R and comes close to the plans of 11 members of the European Union for the implementation of an FTT. We implement the FTT on market LEFT where the market and residence principles are applied at the same time: subjects with home market LEFT are taxed irrespective whether they trade on the left or right market (residence principle). In addition, subjects with home market RIGHT who trade on the LEFT market are taxed as well (market principle). Only trading on the right market remains untaxed for subject with home market RIGHT.

#### 2.1.4 Treatment MR_DIFF_: market and residence principle on different markets

This treatment stands for the possible scenario that one country imposes an FTT according to the residence principle and another country imposes a FTT following the market principle. An FTT for subjects with home market LEFT is applied according to the residence principle. In addition, the market principle is applied on the right market. Thus, subjects with home market LEFT are taxed by their home jurisdiction whenever they trade and additionally face a tax of 0.1% when trading on the market. In contrast, subjects with home market RIGHT are only taxed for trading on their home market, as market LEFT remains untaxed for them.

Table 1 shows the taxation scenarios depending on residence and trading activity, i.e. trading on the left or right market.

**Table 1:** Taxation Scenarios for the Various Treatments Depending on Subjects’ Home Market and Trading Place (LEFT or RIGHT)

	Tax when trading on market…	
	LEFT (%)	RIGHT (%)
Treatment M		
Home Market LEFT	0.1	–
Home Market RIGHT	0.1	–
Treatment R		
Home Market LEFT	0.1	0.1
Home Market RIGHT	–	–
Treatment MR_SAME_		
Home Market LEFT	0.1	0.1
Home Market RIGHT	0.1	–
Treatment MR_DIFF_		
Home Market LEFT	0.1	0.2
Home Market RIGHT	–	0.1

*Notes*. In case of taxation, entries show the tax rate conditional on the residence of the subjects for each market.

### 2.2 The Order of Implementing the FTT

In all treatments, we use a specific taxation scenario either in the first phase (periods 1–4) or in the second phase (periods 5–8). For instance, when we introduce an FTT in the first phase, we abolish the FTT in the second phase. To control for possible learning effects in each treatment, we impose an FTT in half of the sessions in the first phase and, in the second phase, in the other half of the sessions. Before the beginning of each phase subjects are informed about the imposition/abolition of an FTT with an announcement screen. This screen is shown for one minute and outlines in detail how the FTT is levied. It also provides a calculation example for taxation. Subjects do not get any information about the possible implementation of an FTT before the main experiment starts and they are not informed in advance whether and when the taxation is changed again, i.e. the taxation changes come as a surprise. Once an FTT has been introduced, the tax rate is also displayed on the trading screen.

### 2.3 Market Architecture and Implementation

In each session, half of the subjects are initially endowed with 75 units of the asset and 3,000 in Taler (experimental currency). The other half starts with 25 units of the asset and 5,000 in Taler. Given an initial fundamental value FV_0_ of 40, each subject's initial wealth is 6,000 in Taler. Holdings of assets and Taler are carried over from one period to the next. Furthermore, subjects are able to go short up to 100 units of the asset and 6,000 in Taler.^9^ Before the beginning of a new period, all order books are emptied and there are no interest payments on holdings in assets or cash. To avoid end‐of‐experiment effects, subjects are told that the experiment will end between periods 6 and 12 randomly.

In this experiment, all units of the asset are bought back at the fundamental value of the last period. Thus, final wealth is the sum of the portfolio value of the asset (units of the asset held multiplied by the fundamental value of the last period) and cash holdings. This sum is converted into euro at an exchange rate of 1 EUR = 400 Taler.

In total, we conducted 12 sessions for each of the four treatments, resulting in 48 sessions and a total of 480 subjects participating in the experiments. All subjects were economics and business students at the University of Innsbruck, recruited with ORSEE (Greiner, 2004). Sessions were computerised using zTree 3.2.8 (Fischbacher, 2007) and lasted about 90 minutes. Average payment to subjects was EUR 20.4.^10^

### 2.4 Elicitation of Risk Attitude and Loss Aversion

In this experiment, we also conducted two tasks to elicit subjects’ risk attitudes and loss aversion. To test for subjects’ risk attitudes, we employ a mechanism based on Gneezy and Potters (1997). We endow subjects with EUR 2, out of which they can invest an amount X in a 50/50 coin flip lottery. If the subject wins in the lottery, she earns EUR 2 + 1.5X, and if she loses she earns EUR 2 − X. The more risk averse, the less a subject would invest in the lottery, and thus the lower is X.

For the elicitation of loss aversion, we employ a method developed by Gächter *et al*. (2007). Subjects are asked to either accept or reject a series of coin flip lotteries. One of the lotteries is later chosen randomly to determine a subject's earnings. In case, the randomly chosen lottery is rejected, the subject earns EUR 0, regardless of the outcome of the coin flip. In case the lottery is accepted, the subject either earns EUR 5 or loses an amount X. The amount X varies across lotteries, ranging from a minimum loss of EUR 2 to a maximum loss of EUR 6. The row in which a subject switches from accepting the lottery to rejecting it defines the loss aversion parameter. It ranges from ‘larger than 2.5’ in case of rejecting all lotteries to ‘lower than 0.83’ in case of accepting all lotteries.^11^

## 3 The Effects of FTT on Aggregate Market Outcomes

We use the following panel regression model to investigate the consequences of an FTT on the market variables trading volume, price volatility and market efficiency:

(2) $$y_{m,p}=\alpha +\beta_{1}{\mathrm{LEFT}}_{p}+\beta_{2}{\mathrm{RIGHT}}_{p}+\epsilon_{m,p}.$$

Here, *y*_*m*,*p*_ is a generic placeholder for the dependent variables explained below, *m* indicates cross‐section (either the LEFT or RIGHT market in a specific session) and *p* phase (i.e. four periods in which a certain taxation scenario is applied). LEFT is a binary dummy for the left market and RIGHT is a binary dummy for the right market when a taxation scenario is applied. Consequently, intercept *α* represents the state in which both markets are untaxed, i.e. no taxation scenario is imposed. We apply clustered standard errors on a session level to allow for correlation within sessions and independence of observations between sessions. In addition, we run pairwise Wald tests to test for differences between the left and the right market when a taxation scenario is applied.

Table 2 provides formulae for the dependent variables on a macrolevel. Following Kirchler *et al*. (2011), we normalise trading volume (*VOL*) by the mean and standard deviation of trading volume in each session *s* to control for idiosyncratic effects of individual sessions. As one can see from Table 2, the means and standard deviations are calculated from period data. To arrive at the normalised volume of phase *p* of market *m* (either LEFT or RIGHT), the average of the respective four period values is calculated.

**Table 2:** Formulae for the Calculation of Variables on the Market Level

Measure	Calculation
Normalised trading volume	$VOL_{s,m,k}\;=\;\left(vol_{s,m,k}\;-\;\bar{vol_{s}}\right)/\sigma_{s}^{vol}$
Normalised returns (tick data)	$RET_{s,m,i}\;=\;\left(ret_{s,m,i}\;-\;\bar{ret_{s}}\right)/\sigma_{s}^{ret}$
SD of normalised returns	*SDRET* = *SD*(*RET*_*s*,*m*,*i*_)
Relative absolute deviation	$RAD_{s,m,k}\;=\;\left|\bar{P_{s,m,k}}\;-\;FV_{s,m,k}\right|/\bar{\left|FV_{s}\right|}$
Normalised tax revenues	$TAX_{m,k}\;=\;\left(tax_{m,k}\;-\;\bar{tax_{m}}\right)/\sigma_{m}^{tax}$

*Notes. s* = session; *m* = market (either LEFT or RIGHT); *k* = period; *i* = trades. *vol*_*s*,*m*,*k*_ = units of the asset traded in period *k*; $\bar{vol_{s}}$ = average trading volume per period of the asset in session *s*; $\sigma_{s}^{vol}$ = standard deviation of all trading volumes per period of the asset in session *s*;* ret*_*s*,*m*,*i*_ =  ln (*P*_*s*,*m*,*i*_) −  ln (*P*_*s*,*m*,*i*−1_); *P*_*s*,*m*,*i*_ = trading price of trade *i*; $\bar{ret_{s}}$ = average of all returns (*ret*) in session *s*; $\sigma_{s}^{ret}$ = standard deviation of all returns (*ret*) in session *s*; $\bar{P_{s,m,k}}$ = (volume‐weighted) mean price; *FV*_*s*,*m*,*k*_ =  fundamental value in session *s* and period *k* (identical in both markets); $\bar{FV_{s}}$ =  average fundamental value of the session; *tax*_*m*,*k*_ = tax revenues in Taler in market *m* and period *k*; $\bar{tax_{m}}$ =  average tax revenues per period in Taler in market *m*; $\sigma_{m}^{tax}$ = standard deviation of all tax revenues per period in Taler in market *m*.

A similar approach as for normalised trading volume is applied for the volatility measure – the standard deviation of normalised returns (*SDRET*). Log‐returns between consecutive trades *i*,* ret*_*s*,*m*,*i*_, are normalised by the mean and the standard deviation in each session.^12^ The standard deviation of these normalised returns in each market phase serves as the dependent variable. With this approach, sessions with idiosyncratic effects in the absolute level of volatility become comparable.

As a proxy for mispricing, relative absolute deviation (*RAD*) is calculated as the absolute difference between mean prices per period and the respective FVs, benchmarked at the average FV in the market (Stöckl *et al*., 2010). Hence, a high level of *RAD* indicates strong mispricing and therefore a low level of price efficiency.

Additionally, we measure the level of tax revenues (*TAX*) prior to and after the imposition of an FTT. We calculate both, naive hypothetical tax revenues of untaxed markets by multiplying the trading volume with the tax rate and actual realised tax revenues after the imposition of the tax. We further normalise tax revenues (either naive or realised) by the mean and standard deviation in each market. We do not normalise on a session level as we want to measure the impact of a tax on the tax revenues of each individual jurisdiction. Thus, we use a different regression model which is outlined in subsection 3.4.

For the variables *VOL*,* RAD* and *TAX* period values are calculated first and the mean per phase *p* and market *m* is used in the regression.

### 3.1 Trading Volume

**Figure 1:**
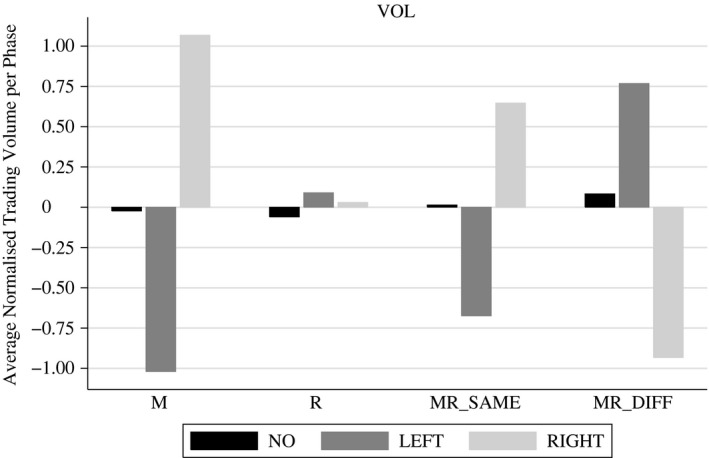
Descriptive Statistics for *VOL (normalised trading volume) Averaged Per Phase and Conditional on Treatment and Taxation Scenario* *Notes. NO* stands for periods without any tax, *LEFT* for the left market and *RIGHT* for the right market when a tax is applied (on any market).

Figure 1 shows descriptive statistics for normalised trading volume (*VOL*) and Table 3 provides the results of the regressions according to (2).

**Table 3:** Trading Volume (VOL) across Treatments

*VOL*	M	R	MR_SAME_	MR_DIFF_
Intercept	−0.023	−0.060	0.014	0.083
	(−0.571)	(−0.592)	(0.136)	(1.186)
LEFT	−0.997***	0.150	−0.688***	0.685***
	(−10.420)	(0.704)	(−2.962)	(3.543)
RIGHT	1.091***	0.090	0.633***	−1.016***
	(13.518)	(0.282)	(2.926)	(−9.338)
*Pairwise Wald tests*:				
LEFT *versus* RIGHT	976.00***	0.03	43.68***	137.90***
*N*	48	48	48	48

*Notes*. Treatments: M: market tax on market LEFT. R: residence tax for residents of market LEFT. MR_SAME_: residence tax for residents of market LEFT and corresponding market tax on market LEFT. MR_DIFF_: residence tax for residents of market LEFT and corresponding market tax on market RIGHT. Variables: Intercept: phase in which both markets are untaxed. LEFT: market LEFT, either taxed or untaxed. RIGHT: market RIGHT, either taxed or untaxed. *, ** and *** represent the 10%, 5% and the 1% significance levels of a double‐sided test. Top: Coefficient values with corresponding z‐values (in parentheses) are provided. Bottom: t‐statistics of pairwise Wald tests are shown.

In treatment M, trading volume drops significantly on the left market (taxed market) and increases significantly on the right market (untaxed market) after an FTT is imposed. This is straightforward, as avoiding the tax is easy for everybody by trading on the untaxed RIGHT market. In contrast, treatment R shows almost no differences in trading volume after a tax is levied on subjects with home market LEFT (residence principle). Thus, we observe no major distorting effects of an FTT when it is implemented according to the residence principle.

Treatment MR_SAME_ shows similar patterns as treatment M, though the effects are somewhat weaker. Again, trading volume is significantly reduced on the left market where an FTT is imposed on residents and as a market tax for foreigners. Trading volume increases significantly on the right market. This pattern is driven by residents of market RIGHT who leave the left market and trade on the right market without any tax burden. However, traders with home market LEFT still provide liquidity to the left market, making the effects less pronounced compared to treatment M. In treatment MR_DIFF_ one can observe the opposite effects: a strong and significant increase in trading volume in the left market and a significant decrease of trading volume in the right market. Subjects with home market LEFT avoid possible double taxation on the right market and subjects with home market RIGHT also shift their trading activity to the left market to avoid taxation on their home market.

### 3.2 Volatility

**Figure 2:**
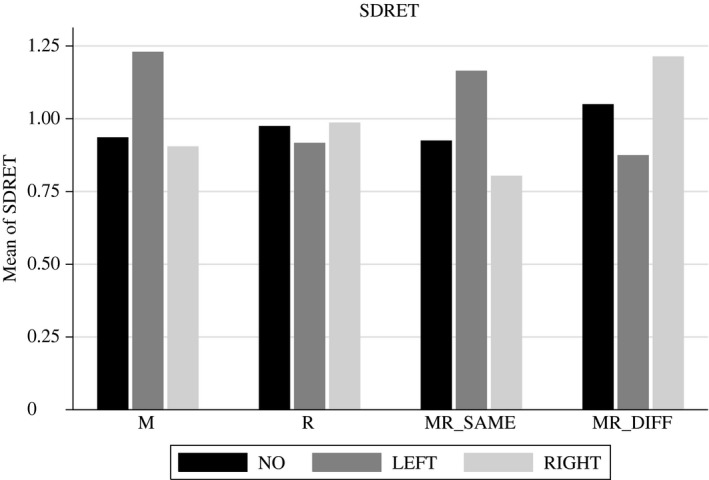
Descriptive Statistics for *SDRET (standard deviation of normalised returns) Averaged Per Phase and Conditional on Treatment and Taxation Scenario* *Notes*. *NO* stands for periods without any tax, *LEFT* for the left market and *RIGHT* for the right market when a taxation scenario is applied.

One of the most controversially discussed issues surrounding the implementation of an FTT is how price volatility is affected. Descriptive results are outlined in Figure 2 and econometric estimations are shown in Table 4.

**Table 4:** Volatility (SDRET) across Treatments

*SDRET*	M	R	MR_SAME_	MR_DIFF_
Intercept	0.935***	0.974***	0.924***	1.049***
	(9.748)	(11.433)	(13.020)	(13.905)
LEFT	0.294	−0.058	0.240	−0.175
	(1.188)	(−0.280)	(1.073)	(−1.137)
RIGHT	−0.031	0.012	−0.121	0.164
	(−0.176)	(0.072)	(−0.883)	(1.060)
*Pairwise Wald tests*:				
LEFT *versus* RIGHT	3.93**	0.68	7.14***	4.81**
*N*	47	48	48	45

*Notes*. Treatments. M: market tax on market LEFT. R: residence tax for residents of market LEFT. MR_SAME_: residence tax for residents of market LEFT and corresponding market tax on market LEFT. MR_DIFF_: residence tax for residents of market LEFT and corresponding market tax on market RIGHT. Variables: Intercept: phase in which both markets are untaxed. LEFT: market LEFT, either taxed or untaxed. RIGHT: market RIGHT, either taxed or untaxed. *, ** and *** represent the 10%, 5% and the 1% significance levels of a double‐sided test. Top: Coefficient values with corresponding z‐values (in parentheses) are provided. Bottom: t‐statistics of pairwise Wald tests are shown.

We find that the development of volatility varies markedly across treatments. After the imposition of the FTT in treatment M, the level of volatility increases in the taxed market (LEFT), whereas it remains almost unchanged in the untaxed market (RIGHT). Most importantly, we report a significant difference between the left market and the right market when a tax is levied (see pairwise Wald tests in Table 4). However, in treatment R, no differences between market LEFT and market RIGHT are visible. Thus, imposing a residence principle on subjects with home market LEFT causes no changes in volatility. Similarly to treatment M, we report an increase of volatility in the left market in treatment MR_SAME_ and a slight decrease in volatility in the right market. Again, we find a significant difference between the left and the right market when a tax is imposed. In treatment MR_DIFF_ we find the opposite pattern: volatility decreases in market LEFT and increases in market RIGHT when a residence tax (LEFT) and a market tax (RIGHT) are applied. Volatility in both markets is significantly different from each other. Hence, volatility in our markets is mostly volume‐driven: whenever volume is high, volatility is low, and *vice versa*.

### 3.3 Price Efficiency

**Figure 3:**
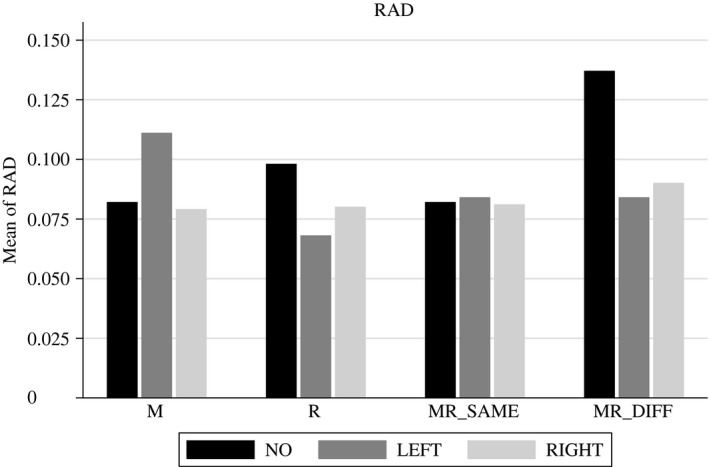
Descriptive Statistics for *RAD (relative absolute deviation of prices compared to fundamentals) Averaged Per Phase and Conditional on Treatment and Taxation Scenario* *Notes*. *NO* stands for periods without any tax, *LEFT* for the left market and *RIGHT* for the right market when a taxation scenario is applied.

The values of *RAD* in the different treatments are shown in Figure 3. Econometric tests are provided in Table 5.

**Table 5:** Price Efficiency (RAD) across Treatments

*RAD*	M	R	MR_SAME_	MR_DIFF_
Intercept	0.082***	0.098***	0.082***	0.137**
	(5.915)	(7.313)	(7.478)	(2.585)
LEFT	0.029	−0.030***	0.002	−0.053
	(0.876)	(−3.021)	(0.114)	(−1.078)
RIGHT	−0.003	−0.018	−0.001	−0.047
	(−0.200)	(−1.413)	(−0.079)	(−0.871)
*Pairwise Wald tests*:				
LEFT *versus* RIGHT	1.35	4.64**	0.46	0.17
*N*	48	48	48	46

*Notes*. Treatments: M: market tax on market LEFT. R: residence tax for residents of market LEFT. MR_SAME_: residence tax for residents of market LEFT and corresponding market tax on market LEFT. MR_DIFF_: residence tax for residents of market LEFT and corresponding market tax on market RIGHT. Variables: Intercept: phase in which both markets are untaxed. LEFT: market LEFT, either taxed or untaxed. RIGHT: market RIGHT, either taxed or untaxed. *, ** and *** represent the 10%, 5% and the 1% significance levels of a double‐sided test. Top: Coefficient values with corresponding z‐values (in parentheses) are provided. Bottom: t‐statistics of pairwise Wald‐tests are shown.

They show that the implementation of an FTT has no significant effect on price efficiency in any of treatments M, MR_SAME_ and MR_DIFF_. Only when a residence tax is levied in treatment R, is mispricing significantly reduced in the left market. This is mainly driven by one outlier in a market that was untaxed. Therefore inefficiency was highest in this treatment. However, the inefficiency observed in this treatment when LEFT is taxed, is at the same level as in the other three treatments. Thus, the reduced inefficiency is a result of a less efficient benchmark, rather when indeed lower inefficiency, when compared to other treatments.

### 3.4 Tax Revenues

**Figure 4:**
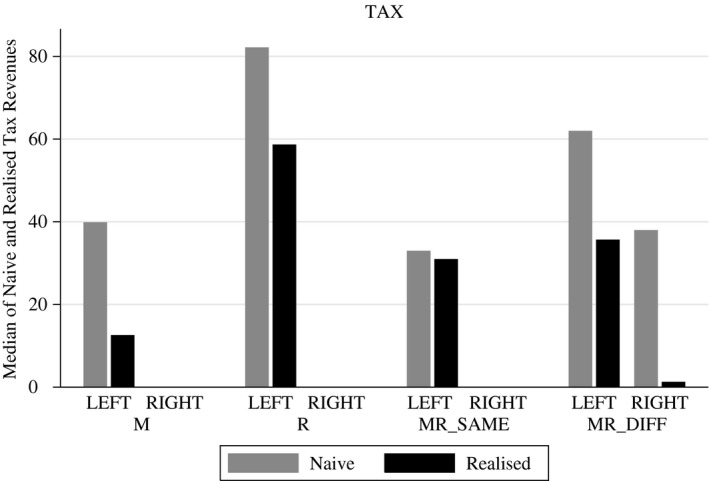
Descriptive Statistics for Naive and Hypothetical Tax Revenues *Notes*. Median of hypothetical and realised tax revenues (in Taler) for market LEFT across the various taxation scenarios and market RIGHT in MR_DIFF_.

In the political debate on the implementation of an FTT, tax revenues are a core argument of the proponents of the tax. Therefore, we calculate a naive estimate of hypothetical tax revenues – i.e. tax revenue if trading volume would not change after the introduction of a tax – and compare it to the actually realised tax revenues in each treatment. Figure 4 gives descriptive results on naive and realised tax revenues.

Since we measure level of tax revenues prior to and after the imposition of an FTT for each market (jurisdiction) separately, a different regression model is used:

(3) $$y_{m,p}=\alpha +\beta_{1}FTT_{p}+\epsilon_{m,p}.$$

Here, *y*_*m*,*p*_ is a generic placeholder for the tax revenues in the phase prior to and after the introduction of an FTT on each market, *m* indicates cross‐section (either the LEFT or RIGHT market in a specific session) and *p* phase (i.e. four periods in which a certain taxation scenario is applied). *FTT* is a binary dummy for the left or right market when a tax is levied and the intercept *α* represents the state in which the market is untaxed.

Table 6 provides the econometric estimation results. We see that in treatment M realised tax revenues are significantly lower than naively estimated hypothetical tax revenues. This result is driven by the strong shift in trading volume out of the taxed market LEFT. In contrast, we find no significant changes in tax revenues prior and after the introduction of an FTT in treatments R and MR_SAME_. The latter effect can be explained by a lower shift in trading volume after the imposition of an FTT as traders taxed according to the residence principle cannot avoid the tax, except by not trading. In treatment MR_DIFF_ market LEFT as well as market RIGHT impose an FTT. Both market places show a significantly lower amount of tax revenues once an FTT is implemented, compared to the naive tax revenues. The extremely low tax revenues in the right market are due to its market tax, which traders avoid by trading on the left market. The significantly lower tax revenues on the left market are triggered by the residence tax of traders with home market LEFT, who trade less. This effect is not compensated by traders with home market RIGHT, although they trade without tax burden on the left market.

**Table 6:** Normalised Tax Revenues (TAX) for Market LEFT and Market RIGHT across the Various Taxation Scenarios

*TAX*	M	R	MR_SAME_	MR_DIFF_	
	LEFT	LEFT	LEFT	LEFT	RIGHT
Intercept	0.625***	0.116	−0.113	0.377***	0.758***
	(7.080)	(0.713)	(−0.729)	(5.721)	(31.506)
FTT	−1.249***	−0.232	0.206	−0.755***	−1.516***
	(−7.080)	(−0.713)	(0.688)	(−5.721)	(−31.506)
*N*	24	24	24	24	24

*Notes*. Treatments: M: market tax on market LEFT. R: tax for residents of market LEFT. MR_SAME_: tax for residents of market LEFT and corresponding market tax on market LEFT. MR_DIFF_: tax for residents of market LEFT and corresponding market tax on market RIGHT. Variables: Intercept: phase in which a market is untaxed. FTT: phase in which a market is taxed. *, ** and *** represent the 10%, 5% and the 1% significance levels of a double‐sided test. Coefficient values with corresponding z‐values (in parentheses) are provided.

### 3.5 Discussion of Market Outcomes

To sum up, the results on a macrolevel show a very clear picture. The implementation of an FTT as a market tax (treatment M) or as a combination of a market and a residence tax (treatment MR_SAME_ and treatment MR_DIFF_) has negative effects on the marketplace which imposes the FTT as a market tax. In particular, subjects avoid a market tax and shift most of their trading volume to the tax haven. Due to the loss of liquidity, volatility is significantly higher in markets with a market tax compared to the ones without market tax. This result is in line with earlier evidence in Hanke *et al*. (2010) and Kirchler *et al*. (2011).

When the residence principle is applied – an institutional form of an FTT not discussed in the literature so far – the affected traders cannot avoid the tax and therefore they provide higher liquidity to the market compared to a scenario with a market tax that is easily avoided. This is also confirmed by running regression (2) with limit orders as dependent variable. The number of posted limit orders decreases significantly in the taxed market after the imposition of an FTT in treatments with a market tax (M and MR_SAME_: z‐values of −7.872 and −4.196 respectively). In contrast, liquidity, measured by the number of limit orders, stays constant (R, z‐value of 0.407) or even increases (MR_DIFF_, z‐value of 6.823) in treatments where a residence tax is imposed without a corresponding market tax. As a consequence, the implementation of an FTT according to the residence principle has no negative effects on volume and volatility in its plain‐vanilla form in treatment R. This non‐negative effect of a residence‐based tax is reinforced and even leads to a significantly lower volatility of this market as soon as the other market imposes a market‐based tax. This pattern is evident in treatment MR_DIFF_ as volatility decreases in the left market because of an inflow of liquidity from the right market.

## 4 Analysis of Individual‐level Data

Our experimental approach allows to examine individual level data on trading behaviour in detail. Here, we investigate whether an FTT has different effects on traders with different levels of risk tolerance. To do so, we first establish whether risk attitudes are related to trading behaviour in general. Then we proceed and check whether an FTT has different effects on traders with different risk attitudes.^13^

### 4.1 Risk Aversion and Individual Trading

To explore differences in the trading behaviour of subjects conditional on their risk attitudes, we run the following regression model:

(4) $$\begin{array}{c} y_{i}=\alpha +\beta_{1}M\times RISK_{i}+\beta_{2}R\times RISK_{i}+\beta_{3}{\text{MR}}_{\mathrm{SAME}}\times RISK_{i}+\beta_{4}{\text{MR}}_{\mathrm{DIFF}}\times RISK_{i}+\epsilon_{i}. \end{array}$$

*y*_*s*_ is a generic placeholder for the dependent variables explained below, *i* identifies a particular subject. The interacted binary dummy variables for each treatment – e.g. M × *RISK* – measure the impact of subject's risk preferences in each treatment. *RISK* stands for the amount X invested in the risky lottery in the risk aversion task (Gneezy and Potters, 1997). The higher a subject's amount X is, the less risk‐averse he is considered. The intercept *α* represents the average of all treatments.^14^

Table 7 presents the dependent variables: normalised trading volume per subject, normalised limit orders per subject and normalised standard deviation of stock holdings per subject.^15^ It is important to mention that as all dependent variables are normalised, the interacted binary dummies only measure the impact of the risk coefficient.

**Table 7:** Formulae for the Calculation of Variables on an Individual Level

Measure	Calculation
Normalised trading volume	$VOL_{i}\;=\;\left(vol_{i}-\bar{vol_{s}}\right)/\sigma_{s}^{vol}$
Normalised limit orders	$LO_{i}\;=\;\left(lo_{i}-\bar{lo_{s}}\right)/\sigma_{s}^{lo}$
Normalised SD of stock holdings	$SDSTOCK_{i}\;=\;\left(sd\_stock_{i}-\bar{sd\_stock_{s}}\right)/\sigma_{s}^{sd\_stock}$

*Notes*. *s* = session; *i* = trader. *vol*_*i*_ = average number of traded assets per period for trader *i*; $\bar{vol_{s}}$ = average trading volume per period in session *s* among all subjects; $\sigma_{s}^{vol}$ = standard deviation of all trading volumes among all subjects in session *s*;* lo*_*i*_ = average number of limit orders per period for trader *i*; $\bar{lo_{s}}$ = average number of of all limit orders (*lo*) among all subjects in session *s*; $\sigma_{s}^{lo}$ = standard deviation of the number of all limit orders (*lo*) in session *s* among all subjects; $sd\_stock_{t}$ = standard deviation of stock holdings per trader *i*; $\bar{sd\_stock_{s}}$ = average standard deviation of stock holdings in session *s*; $\sigma_{s}^{sd\_stock}$ = standard deviation of all standard deviations of stock holdings in session *s*;

Table 8 outlines the results. We find that subjects with high‐risk tolerance coefficients show a significantly higher trading activity. Subjects who are less risk‐averse trade significantly more and post significantly more limit orders compared to their more risk‐averse counterparts. These results are robust across all treatments. As a consequence, subjects with high risk coefficients show a significantly higher standard deviation of stock holdings and therefore hold more volatile and extreme portfolio positions.

**Table 8:** Regression for Differences in Behaviour Conditional on Subjects’ Risk Attitudes

	*VOL*	*LO*	*SDSTOCK*
Intercept	−0.230***	−0.294***	−0.230**
	(−3.499)	(−3.536)	(−2.422)
M × *RISK*	0.159***	0.196***	0.120
	(3.128)	(2.943)	(1.630)
R × *RISK*	0.180***	0.240***	0.207***
	(3.430)	(3.208)	(2.735)
MR_SAME_ × *RISK*	0.199***	0.267***	0.178*
	(2.983)	(3.790)	(1.907)
MR_DIFF_ × *RISK*	0.166***	0.201***	0.199***
	(3.648)	(3.405)	(3.165)
*N*	480	480	480

*Notes*. Treatments: M: market tax on market LEFT. R: residence tax for residents of market LEFT. MR_SAME_: residence tax for residents of market LEFT and corresponding market tax on market LEFT. MR_DIFF_: residence tax for residents of market LEFT and corresponding market tax on market RIGHT. Variables: *VOL*: normalised trading volume. *LO*: normalised limit orders. *SDSTOCK*: normalised standard deviation of stock holdings. Intercept: average across all treatments. *RISK*: amount X invested in the risky lottery in the risk aversion task (Gneezy and Potters, 1997). *, ** and *** represent the 10%, 5% and the 1% significance levels of a double‐sided test. Coefficient values with corresponding z‐values (in parentheses) are provided.

Additionally, we analyse the use of short selling and borrowing cash with regard to subjects’ risk attitudes. As outlined above, short selling and borrowing was allowed up to 100% of the initial endowments in assets and cash. We find that only 64 out of 480 subjects (13.3%) have short positions in assets and 41 subjects (8.5%) have negative cash holdings at the end of at least one period. Approximately, 60% of the subjects who go short in assets or cash at least once have the highest risk coefficient of 2, while only 34% of the subjects who do not use short selling or borrowing have the highest risk coefficient. To test whether there is a significant difference in the distribution of risk coefficients between these two groups, we run a Kolmogorov–Smirnov equality‐of‐distributions test. Indeed, we find a significant difference between the distribution of both groups (D‐value for short selling: 0.2462, p‐value: 0.002, *N* = 480; D‐value for borrowing: 0.2492, p‐value: 0.019; *N* = 480). Thus, the more risk tolerant subjects are, the more they use short selling and borrowing.

We summarise this subsection by noting that subjects’ trading behaviour strongly depends on their risk tolerance. More specifically, subjects with more risk tolerance – i.e. with lower degrees of risk aversion – trade more, post more limit orders, show a higher volatility in their asset holdings and use short selling opportunities more frequently. These effects hold across all treatments. Based on these findings, we can now proceed to answer our final question, whether the imposition of an FTT has different effects on traders with different levels of risk aversion.

### 4.2 Interaction of FTT with Risk Aversion

We apply the following regression model to explore whether subjects with different risk attitudes react differently to the imposition of an FTT:

(5) $$y_{m,p}=\alpha +\beta_{1}RISK_{i}+\epsilon_{i}.$$

Here, *y*_*m*,*p*_ is a generic placeholder for the dependent variables and *RISK*_*i*_ stands for the risk coefficient of subject *i*.^16^

We use the following dependent variables: first, we calculate the normalised sum of all tax payments per subject *i* (*SUMTAX*_*i*_). This allows to test whether subjects with different risk attitudes show a different proneness for paying the tax. Second, we calculate subject *i*'s ratio between the trading volume on the left market and on the right market when a tax is levied (*MARKETSHARE*_*i*_). Third, we compare each subject's change in trading volume prior and after an FTT is applied on both markets (Δ*VOLLEFT* and Δ*VOLRIGHT*). This enables us to investigate whether risk attitude determines behavioural changes after the imposition of an FTT on each market place. Table 9 shows the variables, and Table 10 presents the econometric results. Except for one single case (which lies well in the limits of chance), we find no differences in behaviour of subjects with different risk attitudes when an FTT is applied.

**Table 9:** Formulae for the Calculation of Variables for Trading Behaviour Prior to and After the Imposition of a FTT

Measure	Calculation
Normalised Tax Payments	$SUMTAX_{i,k}\;=\;\left(sumtax_{i,k}\;-\;\bar{sumtax_{s}}\right)/\sigma_{s}^{sumtax}$
Market Share	*MARKETSHARE*_*i*_ = *vol*_LEFT_*i*_/(*vol*_LEFT_*i*_ + *vol*_RIGHT_*i*_)
Change in volume on market LEFT	$\Delta VOLLEFT_{i}\;=\;vol\_{\mathrm{LEFT}}_{i}/\bar{vol\_noTax_{i}}\;-\;1$
Change in volume on market RIGHT	$\Delta VOLRIGHT_{i}\;=\;vol\_{\mathrm{RIGHT}}_{i}/\bar{vol\_noTax_{i}}\;-\;1$

*Notes. s* = session; *i* = trader; *k* = period. $\bar{vol\_LEFT_{i}}$ = trading volume of trader *i* on the left market in case of taxation; $\bar{vol\_RIGHT_{i}}$ = trading volume of trader *i* on the right market in case of taxation; $\bar{vol\_noTax_{i}}$ =average trading volume of trader *i* in market LEFT and RIGHT in case of no taxation;

**Table 10:** Regression for *SUMTAX*,* MARKETSHARE*, Δ*VOLLEFT* and Δ*VOLRIGHT*

	Overall	M	R	MR_SAME_	MR_DIFF_
*SUMTAX*					
Intercept	−0.068	−0.032	−0.147	0.018	−0.126
	(−0.833)	(−0.156)	(−0.912)	(0.109)	(−0.805)
*RISK*	0.052	0.023	0.115	−0.015	0.091
	(0.834)	(0.157)	(0.918)	(−0.109)	(0.804)
*N*	480	120	120	120	120
*MARKETSHARE*					
Intercept	0.445***	0.087**	0.579***	0.308***	0.901***
	(7.281)	(2.786)	(7.086)	(7.005)	(13.404)
*RISK*	0.009	0.041	−0.008	−0.005	−0.019
	(0.323)	(1.765)	(−0.141)	(−0.206)	(−0.435)
*N*	408	119	58	115	116
Δ*VOLLEFT*					
Intercept	0.450	−0.853***	0.159	0.987	1.396***
	(1.225)	(−10.379)	(0.720)	(0.821)	(4.927)
*RISK*	−0.223	0.142**	0.019	−0.799	−0.236
	(−1.031)	(2.305)	(0.118)	(−1.098)	(−1.448)
*N*	472	118	119	116	119
Δ*VOLRIGHT*					
Intercept	1.048	1.039**	0.172	3.222	−0.804***
	(1.480)	(2.984)	(0.411)	(1.379)	(−5.852)
*RISK*	−0.452	−0.094	0.058	−1.505	0.062
	(−1.074)	(−0.537)	(0.171)	(−1.026)	(0.714)
*N*	472	118	119	116	119

*Notes*. Treatments: M: market tax on market LEFT. R: residence tax for residents of market LEFT. MR_SAME_: residence tax for residents of market LEFT and corresponding market tax on market LEFT. MR_DIFF_: residence tax for residents of market LEFT and corresponding market tax on market RIGHT. Variables: *SUMTAX*: normalised sum of all tax payments per subject. *MARKETSHARE*: subject *i*'s ratio between the trading volume on the left market and on the right market when a tax is levied. Δ*VOLLEFT* and Δ*VOLRIGHT*: subject *i*'s change in trading volume (on market LEFT or RIGHT) between phases with and without the tax. *RISK*: amount X invested in the risky lottery in the risk aversion task (Gneezy and Potters, 1997). *, ** and *** represent the 10%, 5% and the 1% significance levels of a double‐sided test. Coefficient values with corresponding z‐values (in parentheses) are provided.

In sum, this final subsection has provided strong evidence that traders with different risk attitudes do not react differently to the imposition of an FTT. This means that risk tolerant and risk averse traders adapt their trading behaviour in the same way when an FTT is levied. As a consequence, the macroresults of our article are not primarily driven by the tax avoiding behaviour of traders with especially low or high levels of risk aversion. Instead, results on a macrolevel are driven by adaptive behaviour of all traders, which is independent of their risk attitudes (and also independent of their level of loss aversion, as shown in the online Appendix).

## 5 Conclusion

The possible introduction of an FTT in 11 member states of the European Union in 2015 constitutes a very large‐scale policy experiment, with unclear consequences for financial markets all over Europe (and most likely elsewhere). We consider laboratory experiments as ideal, cheap and practically risk‐free testbeds to explore likely consequences of a legislative change before this change is actually implemented.^17^ For this reason, we conduct experiments to explore the effects of an FTT on market outcomes and individual traders. We compare the ‘market principle’ and the ‘residence principle’ as basis of an FTT, examining both principles separately and jointly. We find that applying only the residence principle as basis for an FTT had no significant effects on trading volume or volatility. The market principle, however, results in large and significant shifts in trading volume from the taxed market to the untaxed alternative. With liquidity in the taxed market evaporating, volatility increases significantly, while it drops in the untaxed alternative.

The combined implementation of market and residence principle within one jurisdiction show the following effects: a significant drop in trading volume in the jurisdiction implementing both principles and a respective increase in the other one. By contrast, volatility increases in the jurisdiction with a tax on residents and market tax for foreigners, whereas it drops in the one without any tax burdens. However, both effects are considerably weaker than when only the market principle is applied. This means that adding the residence principle dampens (rather than exacerbates) the negative repercussions from applying a market principle. We consider the latter a particularly interesting, and novel, finding of our experiment. Our results highlight that details of the implementation are of paramount importance and economists should get their hands dirty with these details.

We are aware that any experimental study – even though it is a cheap means of testing behavioural responses to intended policy changes – has its limitations. In our case, it is important to stress that we do not test for allocative efficiency and that we do not explore the issue of risk‐sharing. Also, in our design taxes can be avoided by not trading (or trading less) when the market principle applies but, other than that, there are no loopholes or ways to circumvent the tax. Once we allow for tax evasion, it is no longer clear whether the effects of a market tax would be as strong as they were in our experiment. The permanent fight of national governments against tax evasion shows that, in reality, it is highly likely that tax evasion will continue to prevail even after the introduction of an FTT. Likewise, the application of a residence principle can work properly only if the respective jurisdiction has proper and complete access to a trader's activities. Yet, it is not clear whether foreign market places will always bother to inform a country applying the residence principle when one of its residents trades. So, the ultimate proof of the pudding (the FTT) is in the eating (its actual implementation) but the current article has given a taste of the pudding's likely flavour.

## Supporting information


**Appendix A.** Loss Aversion and Individual Trading.
**Appendix B.** Interaction of FTT with Loss Aversion.
**Appendix C.** Instructions for the Experiments.

**Data S1.**

## Notes

1 The 11 countries are Austria, Belgium, Estonia, France, Germany, Greece, Italy, Portugal, Slovakia, Slovenia and Spain. The level of the FTT is likely to be 0.1% on stock transactions and 0.01% on derivatives transactions. The EU Commission, expecting that stock transactions will fall by 15% and derivative transactions by 75%, still forecasts raising 30 to 35 bn euro in tax income per year.

2 See ul Haq *et al*. (1996), Spahn (2002), Habermeier and Kirilenko (2003) and McCulloch and Pacillo (2011) for various surveys.

3 See Kupiec (1995), Jones and Seguin (1997), Baltagi *et al*. (2006) and Hau (2006) for more empirical research in this tradition.

4 See Lux (1998), Lux and Marchesi (2000) and Hommes (2006) for studies with the chartist and fundamentalist approach.

5 The relationship between market liquidity and the price impact of orders has also been explored by Ehrenstein *et al*. (2005), Lillo and Farmer (2005) and Mannaro *et al*. (2008). Ehrenstein *et al*. (2005) and Mannaro *et al*. (2008) argue that transaction taxes might have a negative impact on market liquidity, hinting at increasing volatility when the market is illiquid.

6 In particular:

$${\text{FV}}_{k}\left(pathII\right)=80-{\text{FV}}_{k}\left(pathI\right).$$

7 This was implemented by drawing positive estimation errors for half of the subjects and using the respective negative error terms for the other half of subjects.

8 Before trading started subjects had 15 minutes to read written instructions. Questions were answered privately. Then the trading screen was explained and two trial periods (not relevant for payment) were conducted to allow subjects to become familiar with the trading screen and the trading procedure (see online Appendix C for the trading screen and the experimental instructions).

9 The maximum levels of shorting assets and cash add up to double their initial average endowments.

10 Softwares and data sets are available online.

11 If losses were incurred in one part of the experiment they were deducted from profits in other parts. No subject came close to an overall loss.

12 See the discussion in Plerou *et al*. (1999) on the importance of normalising returns from different observations.

13 Since not only risk attitudes might be important for trading on markets, we also consider loss aversion as a potentially important trader characteristic in order to explain trading behaviour. However, in our analysis, we do not observe any significant impact of subjects’ level of loss aversion (parameter *λ*) on their trading behaviour, as shown in online Appendices A and B. In fact, adding loss aversion as an explanatory variable makes the model fit worse (as measured by BIC or AIC). For this reason and for the sake of brevity and readability, we, therefore, relegate the analysis including loss aversion to the online Appendix.

14 We apply clustered standard errors on a session level to allow for correlation within sessions and independence between sessions.

15 Again, we normalise trading volume, limit orders and standard deviation of stock holdings per subject to control for idiosyncratic effects of individual sessions as argued in Section 3.

16 Again, we apply clustered standard errors on a session level.

17 Several researchers have advocated the potential usefulness of experiments for addressing policy‐relevant questions. For instance, Roth (2002) discusses the role of (experimental) economists as institutional engineers. However, it is clear that experiments are by necessity always a simplification and can thus not give a perfect and fully comprehensive picture of the real‐world situation under consideration (see e.g. List (2011) for limitations of laboratory experiments).
