# Supplementary material for: Market versus Residence Principle: Experimental Evidence on the Effects of a Financial Transaction Tax
Source: Econ J (London). 2017 Oct 24;127(605):F610–31. doi: 10.1111/ecoj.12339 (PMC5698715; doi:10.1111/ecoj.12339)
Supplement: Supplementary file 1 — Appendix A. Loss Aversion and Individual Trading. Appendix B. Interaction of FTT with Loss Aversion. Appendix C. Instructions for the Experiments. [file ECOJ-127-F610-s001.pdf]

The Economic Journal, Doi: 10.1111/ecoj.12339 © 2017 The Authors. The Economic Journal published by John Wiley & Sons Ltd on behalf of Royal Economic Society. Published by John Wiley & Sons, 9600 Garsington Road, Oxford OX4 2DQ, UK and 350 Main Street, Malden, MA 02148, USA.

Technical Appendix to

MARKET *VERSUS* RESIDENCE PRINCIPLE: EXPERIMENTAL EVIDENCE ON THE EFFECTS OF A FINANCIAL TRANSACTION TAX

Jürgen Huber, Michael Kirchler, Daniel Kleinlercher and Matthias Sutter

Economic Journal, doi: 10.1111/ecoj.12339

Appendix A. Loss Aversion and Individual Trading

To explore whether we observe differences in the trading behaviour of subjects conditional on their level of loss aversion, we run the following regression model:

$$y_i = \alpha + \beta_1 \mathrm{M} \times LOSS_i + \beta_2 \mathrm{R} \times LOSS_i + \beta_3 \mathrm{MR}_{\mathrm{SAME}} \times LOSS_i + \beta_4 \mathrm{MR}_{\mathrm{DIFF}} \times LOSS_i + \epsilon_i. \quad \text{(A.1)}$$

y_s is a generic placeholder for the dependent variables explained below, i stands for subject. The interacted binary dummy variables for each treatment – e.g. M × *LOSS* – measure the impact of subjects' loss aversion in each treatment. *LOSS* stands for the individual loss aversion parameter λ (Gächter *et al.*, 2007) ranging from larger than 2.5 in case of rejecting all lotteries to smaller than 0.83 in case of accepting all lotteries. The higher the individual loss parameter λ, the more loss averse a subject is. The intercept α represents the average of all treatments. Again, we apply clustered standard errors on a session level to allow for correlation within sessions and independence between sessions. It is important to mention that as all dependent variables are

Table A1

Regression for Differences in Behaviour Conditional on Subjects' Loss Aversion

	VOL	*LO*	*SDSTOCK*
Intercept	0.321**	0.131	−0.050
	(2.013)	(1.067)	(−0.348)
M × *LOSS*	−0.185**	−0.064	0.017
	(−2.272)	(−0.996)	(0.214)
R × *LOSS*	−0.171**	−0.064	0.016
	(−2.150)	(−1.087)	(0.211)
$\mathrm{MR}_{\mathrm{SAME}}$ × *LOSS*	−0.137	−0.055	0.062
	(−1.539)	(−0.824)	(0.820)
$\mathrm{MR}_{\mathrm{DIFF}}$ × *LOSS*	−0.184**	−0.078	0.017
	(−2.227)	(−1.147)	(0.219)
N	458	458	458

Notes. Treatments: M: market tax on market LEFT. R: residence tax for residents of market LEFT. $\mathrm{MR}_{\mathrm{SAME}}$: residence tax for residents of market LEFT and corresponding market tax on market LEFT. $\mathrm{MR}_{\mathrm{DIFF}}$: residence tax for residents of market LEFT and corresponding market tax on market RIGHT. Variables: *VOL*: normalised trading volume. *LO*: normalised limit orders. *SDSTOCK*: normalised standard deviation of stock holdings. Intercept: average across all treatments. *LOSS*: individual loss aversion parameter λ. *, ** and *** represent the 10%, 5% and the 1% significance levels of a double-sided test. Coefficient values with corresponding z-values (in parentheses) are provided.

Table A2

Regression for Differences in Behaviour Conditional on Subjects' Risk and Loss Aversion

	VOL	*LO*	*SDSTOCK*
Intecept	0.090	−0.237	−0.342*
	(0.404)	(−1.311)	(−1.885)
M × *RISK*	0.097	0.158	0.007
	(1.151)	(1.323)	(0.057)
R × *RISK*	0.162**	0.225*	0.259**
	(2.212)	(1.821)	(2.317)
MR_{SAME} × *RISK*	0.099	0.265***	0.094
	(0.885)	(3.120)	(0.676)
MR_{DIFF} × *RISK*	0.168**	0.199**	0.296***
	(2.262)	(2.069)	(4.624)
M × *LOSS*	−0.137	0.011	0.152
	(−1.547)	(0.126)	(1.338)
R × *LOSS*	−0.157*	−0.022	0.004
	(−1.764)	(−0.291)	(0.040)
MR_{SAME} × *LOSS*	−0.081	−0.024	0.150
	(−0.723)	(−0.302)	(1.558)
MR_{DIFF} × *LOSS*	−0.183*	−0.032	−0.034
	(−1.804)	(−0.326)	(−0.392)
N	458	458	458

Notes. Treatments: M: market tax on market LEFT. R: residence tax for residents of market LEFT. MR_{SAME}: residence tax for residents of market LEFT and corresponding market tax on market LEFT. MR_{DIFF}: residence tax for residents of market LEFT and corresponding market tax on market RIGHT. Variables: *VOL*: normalised trading volume. *LO*: normalised limit orders. *SDSTOCK*: standard deviation of stock holdings. Intercept: average across all treatments. *RISK*: amount X invested in the risky lottery in the risk aversion task (Gneezy and Potters, 1997). *LOSS*: individual loss aversion parameter λ. *, ** and *** represent the 10%, 5% and the 1% significance levels of a double-sided test. Coefficient values with corresponding z-values (in parentheses) are provided.

normalised the interacted binary dummies only measure the impact of the loss aversion coefficient.

Table A1 shows that loss aversion has an effect on trading volume in the expected direction. More loss averse subjects trade less. Adding the coefficient of *RISK* to the specification in Table A1, we see from Table A2 that the significance of loss aversion gets weaker and partly insignificant, when we control for *RISK*. In fact, the best model fit (according to BIC and AIC) is given when we only control for *RISK*, as has been done in the main text in Table 8.

Appendix B. Interaction of FTT with Loss Aversion

We apply the following regression model to explore whether subjects with different levels of loss aversion react differently to the imposition of an FTT:

$$y_{m,p} = \alpha + \beta_1 LOSS_i + \epsilon_i. \quad \text{(B.1)}$$

Here, $y_{m,p}$ is a generic placeholder for the dependent variables and $LOSS_i$ stands for the loss aversion coefficient of subject i.[1] Table B1 shows that loss aversion is never significant. This remains true if one adds *RISK* to the specification. Table B2 shows that loss aversion remains insignificant when risk aversion is controlled for. Again, the best model fit (according to BIC and AIC) is given when we only control for *RISK*, as has been done in the main text in Table 10.

[1] Again, we apply clustered standard errors on a session level.

© 2017 The Authors.
The Economic Journal published by John Wiley & Sons Ltd on behalf of Royal Economic Society.

Table B1

Regression for SUMTAX, MARKETSHARE, VOLLEFT and VOLRIGHT

	Overall	M	R	MR_{SAME}	MR_{DIFF}
SUMTAX					
Intercept	0.008	−0.197	0.167	0.025	0.012
	(0.052)	(−0.816)	(0.599)	(0.078)	(0.035)
LOSS	−0.008	0.093	−0.080	−0.005	−0.026
	(−0.103)	(0.731)	(−0.557)	(−0.029)	(−0.141)
N	458	113	115	114	116
MARKETSHARE					
Intercept	0.391***	0.182**	0.461***	0.279***	0.780***
	(6.137)	(2.688)	(4.463)	(3.453)	(9.996)
LOSS	0.035	−0.024	0.050	0.017	0.049
	(1.418)	(−0.968)	(1.215)	(0.456)	(1.525)
N	389	112	56	109	112
Δ*VOLLEFT*					
Intercept	−0.019	−0.494**	0.312	−0.893	1.053**
	(−0.072)	(−2.482)	(1.018)	(−1.105)	(2.324)
LOSS	0.087	−0.090	−0.065	0.506	−0.039
	(0.592)	(−1.135)	(−0.506)	(0.899)	(−0.195)
N	450	111	114	110	115
Δ*VOLRIGHT*					
Intercept	0.140	0.944*	0.697	−0.961	−0.502**
	(0.312)	(2.068)	(0.873)	(−0.543)	(−2.329)
LOSS	0.164	−0.002	−0.219	1.251	−0.119
	(0.566)	(−0.011)	(−0.672)	(1.017)	(−1.127)
N	450	111	114	110	115

Notes. Treatments: M: market tax on market LEFT. R: residence tax for residents of market LEFT. MR_{SAME}: residence tax for residents of market LEFT and corresponding market tax on market LEFT. MR_{DIFF}: residence tax for residents of market LEFT and corresponding market tax on market RIGHT. Variables: *SUMTAX*: normalised sum of all tax payments per subject. *MARKETSHARE*: subject *i*'s ratio between the trading volume on the left market and on the right market when a tax is levied. Δ*VOLLEFT* and Δ*VOLRIGHT*: subject *i*'s change in trading volume prior and after an FTT is applied on both markets. *LOSS*: individual loss aversion parameter λ. *, ** and *** represent the 10%, 5% and the 1% significance levels of a double-sided test. Coefficient values with corresponding z-values (in parentheses) are provided.

© 2017 The Authors.
The Economic Journal published by John Wiley & Sons Ltd on behalf of Royal Economic Society.

Table B2

Regression for SUMTAX, MARKETSHARE, VOLLEFT and VOLRIGHT

	Overall	M	R	MR_{SAME}	MR_{DIFF}
SUMTAX					
Intercept	−0.048	−0.158	−0.036	0.077	−0.131
	(−0.238)	(−0.439)	(−0.092)	(0.153)	(−0.308)
RISK	0.032	−0.022	0.121	−0.030	0.080
	(0.477)	(−0.145)	(0.885)	(−0.197)	(0.644)
LOSS	−0.001	0.088	−0.055	−0.013	−0.009
	(−0.010)	(0.667)	(−0.364)	(−0.067)	(−0.051)
N	458	113	115	114	116
MARKETSHARE					
Intercept	0.355***	0.138*	0.446***	0.274**	0.802***
	(4.516)	(2.054)	(3.948)	(2.854)	(7.258)
RISK	0.021	0.025	0.008	0.003	−0.012
	(0.743)	(1.100)	(0.140)	(0.113)	(−0.266)
LOSS	0.040	−0.019	0.053	0.017	0.047
	(1.551)	(−0.749)	(1.478)	(0.455)	(1.390)
N	389	112	56	109	112
Δ*VOLLEFT*					
Intercept	0.287	−0.688***	0.284	0.388	1.271**
	(0.862)	(−4.281)	(1.011)	(0.424)	(2.652)
RISK	−0.178	0.114*	0.017	−0.738	−0.122
	(−0.847)	(1.862)	(0.107)	(−1.093)	(−0.674)
LOSS	0.049	−0.071	−0.061	0.293	−0.063
	(0.418)	(−0.890)	(−0.549)	(0.757)	(−0.351)
N	450	111	114	110	115
Δ*VOLRIGHT*					
Intercept	0.916**	1.060	0.680	1.347	−0.623*
	(2.088)	(1.481)	(1.240)	(1.399)	(−1.857)
RISK	−0.451	−0.068	0.010	−1.329	0.068
	(−1.127)	(−0.344)	(0.033)	(−1.031)	(0.742)
LOSS	0.067	−0.014	−0.217	0.868	−0.105
	(0.319)	(−0.060)	(−0.770)	(1.086)	(−0.874)
N	450	111	114	110	115

Notes. Treatments: M: market tax on market LEFT. R: residence tax for residents of market LEFT. MR_{SAME}: residence tax for residents of market LEFT and corresponding market tax on market LEFT. MR_{DIFF}: residence tax for residents of market LEFT and corresponding market tax on market RIGHT. Variables: *SUMTAX*: normalised sum of all tax payments per subject. *MARKETSHARE*: subject *i*'s ratio between the trading volume on the left market and on the right market when a tax is levied. Δ*VOLLEFT* and Δ*VOLRIGHT*: subject *i*'s change in trading volume prior and after an FTT is applied on both markets. *RISK*: amount X invested in the risky lottery in the risk aversion task (Gneezy and Potters, 1997). *LOSS*: individual loss aversion parameter λ. *, ** and *** represent the 10%, 5% and the 1% significance levels of a double-sided test. Coefficient values with corresponding z-values (in parentheses) are provided.

© 2017 The Authors.
The Economic Journal published by John Wiley & Sons Ltd on behalf of Royal Economic Society.

Appendix C. Instructions for the Experiments

C.1. *Background of the Experiment*

This experiment is concerned with replicating an asset market where 10 traders can trade one asset on two different marketplace (Market LEFT and market RIGHT) simultaneously. Thereby one half of the subjects is a resident of market LEFT and the other half of market RIGHT. You are a resident of market XY – this will be displayed on the trading screen as well.

C.2. *Market Properties*

- Initial endowment: half of the traders start with 75 units of the asset and 3,000 cash, while the other half of the traders start with 25 units of the asset and 5,000 cash.
- There are two markets where the asset can be traded – markets LEFT and RIGHT.
- No interest is paid.
- The prices in the two markets can deviate.

C.3. *Fundamental Value of the Asset*

The fundamentally justified value – fundamental value need not equal the price – of the asset (expressed in cash) is the value that would result from a full and fair analysis of the asset. In reality it depends on micro and macroeconomic variables. In our market the fundamental the asset (expressed in cash) is modelled as a stochastic process:

$$\mathrm{FV}_k = \mathrm{FV}_{k-1} \times e^{\gamma_k},$$

where FV_k stands for the fundamental value in period k and γ_k is a normally distributed random variable with a mean of zero and a standard deviation of 10%. The fundamental value in the current period (increased by 0.5%) it thus the best estimate of the fundamental value in the next period.

C.4. *Information on the Fundamental Value of the Asset*

Each period each subject receives a private signal (SIGNAL) on the fundamental value of the asset (expressed in cash). This signal can be above or below the actual fundamental value with equal probability. Most signals are close to the true fundamental value, as only an error term with an expected value of zero and a standard deviation of 5% is added to the fundamental value.

C.5. *Trading*

- All subjects can buy and sell units of the asset at any time. This can be done on the LEFT or RIGHT market – switching between markets is free and causes no extra costs. Short selling (negative holdings) is possible up to an amount of −100 units of the asset and −6.000 in cash. The volume of each transaction is limited to 20 units of the asset but trading volume within a period is unlimited.
- Each period subjects can enter as many BIDs and ASKs (between 1 and 999) as they want – again without restrictions on the LEFT and RIGHT market.
- IMPORTANT: The price of the asset is set exclusively by you and the other subjects in the market by supply and demand.

© 2017 The Authors.
The Economic Journal published by John Wiley & Sons Ltd on behalf of Royal Economic Society.

C.6. *Calculating Wealth during the Experiment*

Your wealth (expressed in cash) during the experiment comprised the value of your holdings in the asset (units of the asset multiplied by the last price) plus the holdings in cash. For valuing the asset the last price is used:

$$\text{Wealth} = (\text{units of the asset} \times \text{price of the asset}) + \text{cash}.$$

If prices in the two markets deviate, the current price with the higher trading volume is used.

C.7. *Payout in EUR in the End of the Experiment*

Your payment in euro depends on your total wealth at the end of the experiment. Your holdings of the asset will be valued at their fundamental value (not price!) of the last period. The final payment is calculated as follows:

$$\text{Final wealth} = (\text{units of the asset} \times \text{fundamental value}) + \text{cash},$$
$$\text{Payout in EUR} = \text{Final wealth}/400.$$

Example: assets: 30, fundamental value of the asset: 45, cash: 5,050.

$$\text{Final wealth} = (30 \times 45) + 5,050 = 6,400$$
$$\text{Payout in EUR} = 6,400/400 = 16 \text{ euro}.$$

The trading screen looks as follows:[2]

[2] Please note that we have chosen the left market to be the HOME MARKET in this example screenshot.

© 2017 The Authors.
The Economic Journal published by John Wiley & Sons Ltd on behalf of Royal Economic Society.

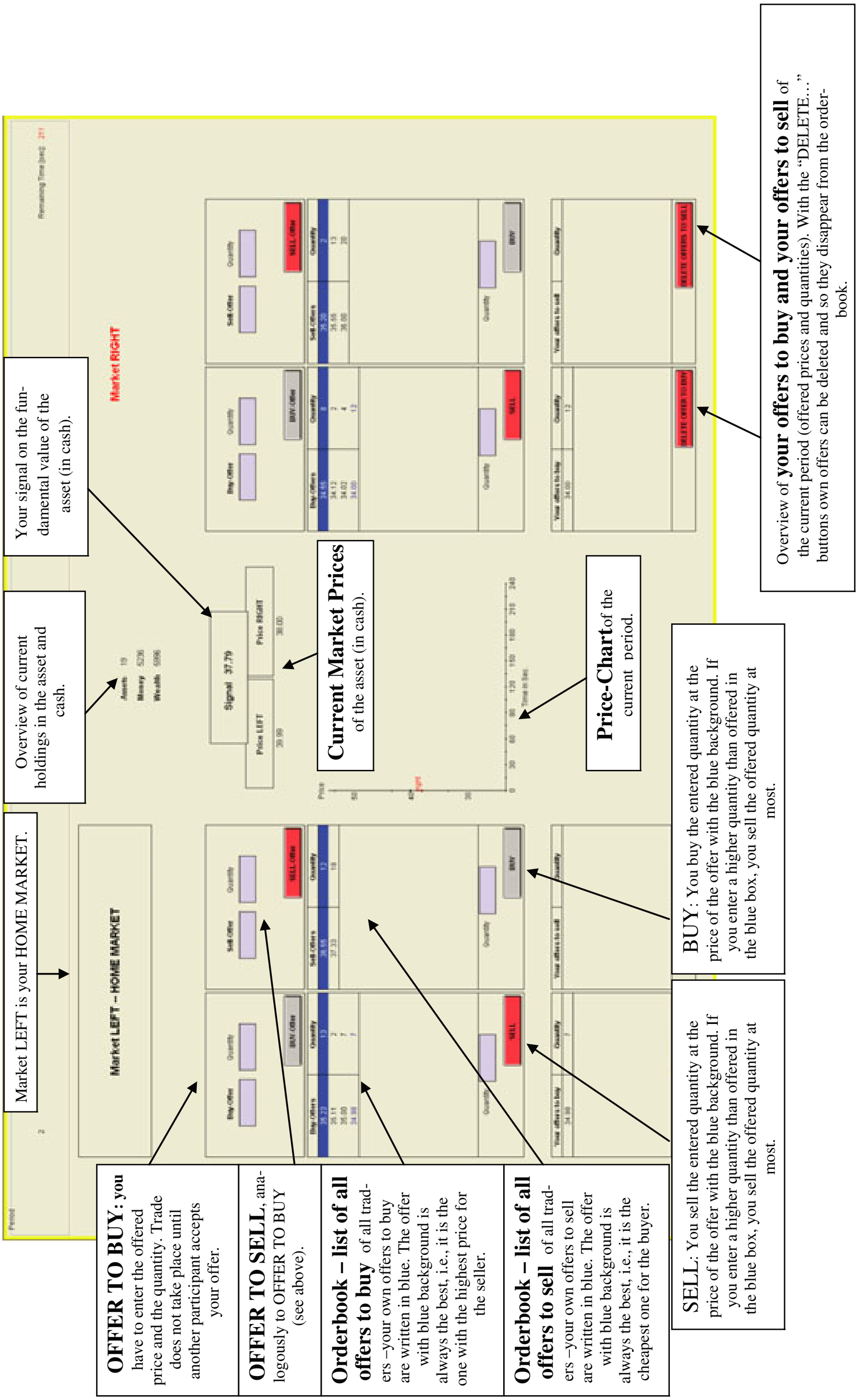

© 2017 The Authors.

C.8. *Important Details*

- Each trading period lasts 240 seconds, i.e. four minutes.
- The experiment lasts between 6 and 12 periods.

After each trading period a history screen is shown for 10 seconds to provide you with information on what happened in the market:

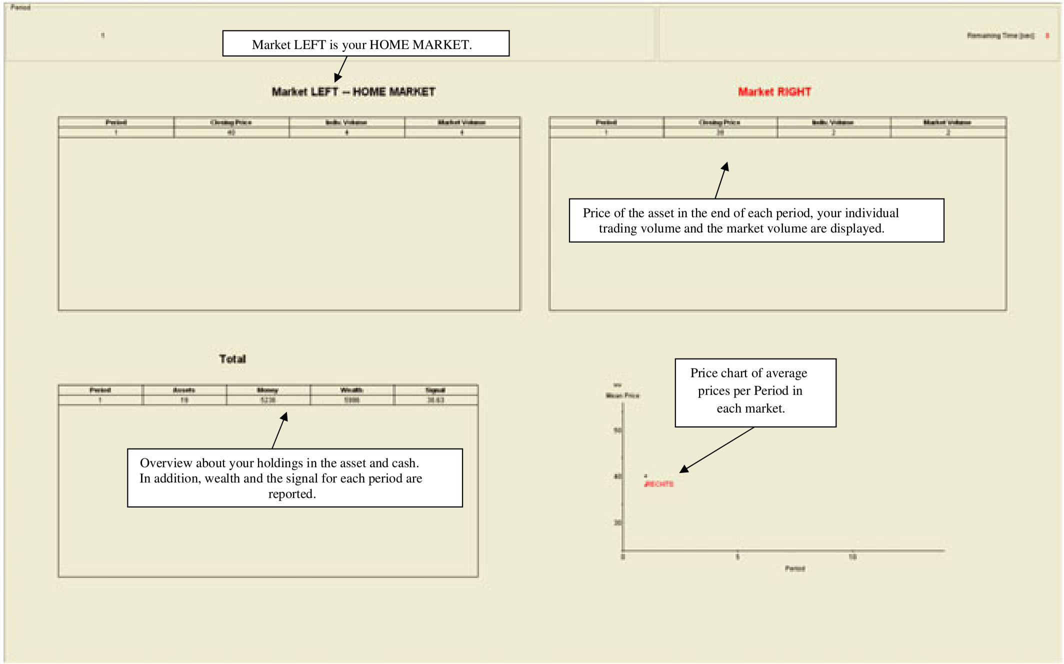

© 2017 The Authors.
The Economic Journal published by John Wiley & Sons Ltd on behalf of Royal Economic Society.

Reference

Gächter, S., Johnson, E.J. and Hermann, A. (2007). 'Individual-level loss aversion in riskless and risky choices', CeDEx Discussion Paper No. 2007-02.

© 2017 The Authors.
The Economic Journal published by John Wiley & Sons Ltd on behalf of Royal Economic Society.
