# Supplementary material for: Market versus Residence Principle: Experimental Evidence on the Effects of a Financial Transaction Tax
Source: Econ J (London). 2017 Oct 24;127(605):F610–31. doi: 10.1111/ecoj.12339 (PMC5698715; doi:10.1111/ecoj.12339)

**Liebe/r Teilnehmer/in**
**Wir möchten Sie im Rahmen des Experiments herzlich begrüßen und bitten Sie von nun an nur mehr mit den Experiment-Leitern zu sprechen.**

---

## Hintergrund des Experiments

Beim vorliegenden Experiment handelt es sich um die Nachbildung von Wertpapiermärkten, auf denen 10 Marktteilnehmer in *k* aufeinander folgenden Perioden ein Wertpapier auf zwei unterschiedlichen Handelsplätzen (Markt LINKS, Markt RECHTS) handeln. Dabei ist die Hälfte der Händler auf dem linken Markt beheimatet (Heimatmarkt = Markt LINKS) und die andere Hälfte auf dem rechten Markt (Heimatmarkt = Markt RECHTS). Ihr Heimatmarkt ist der Markt LINKS – dies ist auch auf dem Handelsbildschirm gekennzeichnet.

## Merkmale der Märkte

- Anfangsausstattungen: Die Hälfte der Marktteilnehmer startet mit ***75 Wertpapieren*** und ***3000 Bargeld***, die andere Hälfte mit ***25 Wertpapieren*** und ***5000 Bargeld***.
- Es gibt 2 Märkte, auf denen gehandelt werden kann - LINKS (Ihr Heimatmarkt) und RECHTS.
- Geldbestände werden nicht verzinst.
- Die Preise auf den Märkten können sich unterscheiden.

## Fundamentalwert des Wertpapiers

Der fundamental gerechtfertigte Wert – ***Fundamentalwert*** ≠ Kurs (Preis) – des Wertpapiers ist jener Wert der sich bei einer fairen Bewertung des Wertpapiers ergibt. Er ändert sich von Periode zu Periode zufällig, wobei er mit gleicher Wahrscheinlichkeit steigt oder fällt:

$$P_k = P_{k-1} + \varepsilon \ ,$$

wobei $P_k$ den Fundamentalwert des Wertpapies in Periode $k$ kennzeichnet und $\varepsilon$ einen normalverteilten Zufallsterm mit einem Erwartungswert von Null und einer Standardabweichung von 10% darstellt. Der Fundamentalwert dieser Periode ist somit der beste Schätzer für den Fundamentalwert in der darauf folgenden Periode.

## Schätzung des Fundamentalwerts des Wertpapiers

Da kein Teilnehmer den exakten Fundamentalwert des Wertpapiers kennt, erhalten Sie in jeder Runde lediglich eine Schätzung (SIGNAL) des Fundamentalwerts. Dieses Signal weicht vom tatsächlichen Fundamentalwert positiv oder negativ ab. Der Großteil der Signale weicht nur geringfügig vom Fundamentalwert ab, größere Abweichungen sind aber möglich. Die Schätzungen (SIGNALE) schwanken mit einer Standardabweichung von 5% um den tatsächlichen Fundamentalwert, wobei ihre Schätzung in einer Periode unabhängig von den Schätzungen in allen anderen Perioden ist.

**Zusammensetzung des Gesamtvermögens während des Experiments**

Während des Experiments setzt sich ihr Gesamtvermögen wie folgt zusammen: Ihr ***Vermögen in Wertpapieren*** (Stück x aktueller Kurs) plus ihr ***Bestand an Bargeld***. Zur Bewertung ihres Vermögens in Wertpapieren wird der jeweilige aktuelle Kurs (Preis) verwendet.

Vermögen = (Stück * Kurs) + Bargeld

Wenn die Kurse auf beiden Märkten voneinander abweichen, wird zur laufenden Bewertung des Vermögens immer jener Kurs herangezogen, bei dem bei der letzten Transaktion mehr Volumen gehandelt wurde.

**Auszahlung in EUR am Ende des Experiments**

Ihre ***Auszahlung in EUR*** richtet sich nach der Höhe Ihres Vermögens am Ende des Experiments. Die Bestände von Wertpapier A werden dabei mit dem ***Fundamentalwert*** (NICHT mit dem aktuellen Kurs!) der letzten Periode bewertet. Im Anschluss wird Ihre Auszahlung in EUR folgendermaßen berechnet:

Endvermögen = Bestand Wertpapier * Fundamentalwert + Bestand Bargeld;
Auszahlung = Endvermögen/400

Bsp: Bestand Wertpapier: 30; Fundamentalwert des Wertpapiers am Ende des Experiments: 45; Bestand Bargeld: 5050.

Endvermögen = 30 * 45 + 5050 = 6400
Auszahlung = 6400/400 = EUR 16

**Wichtige Details**

- Jede Handelsperiode dauert 240 Sekunden, d.h. 4 Minuten.
- Das Experiment endet zufällig zwischen 6 und 12 Perioden.
- Als Kommazeichen muss der Punkt (.) verwendet werden.

Der Handelsschirm sieht wie folgt aus (beachten Sie, dass der Aufbau für beide Märkte identisch ist!):

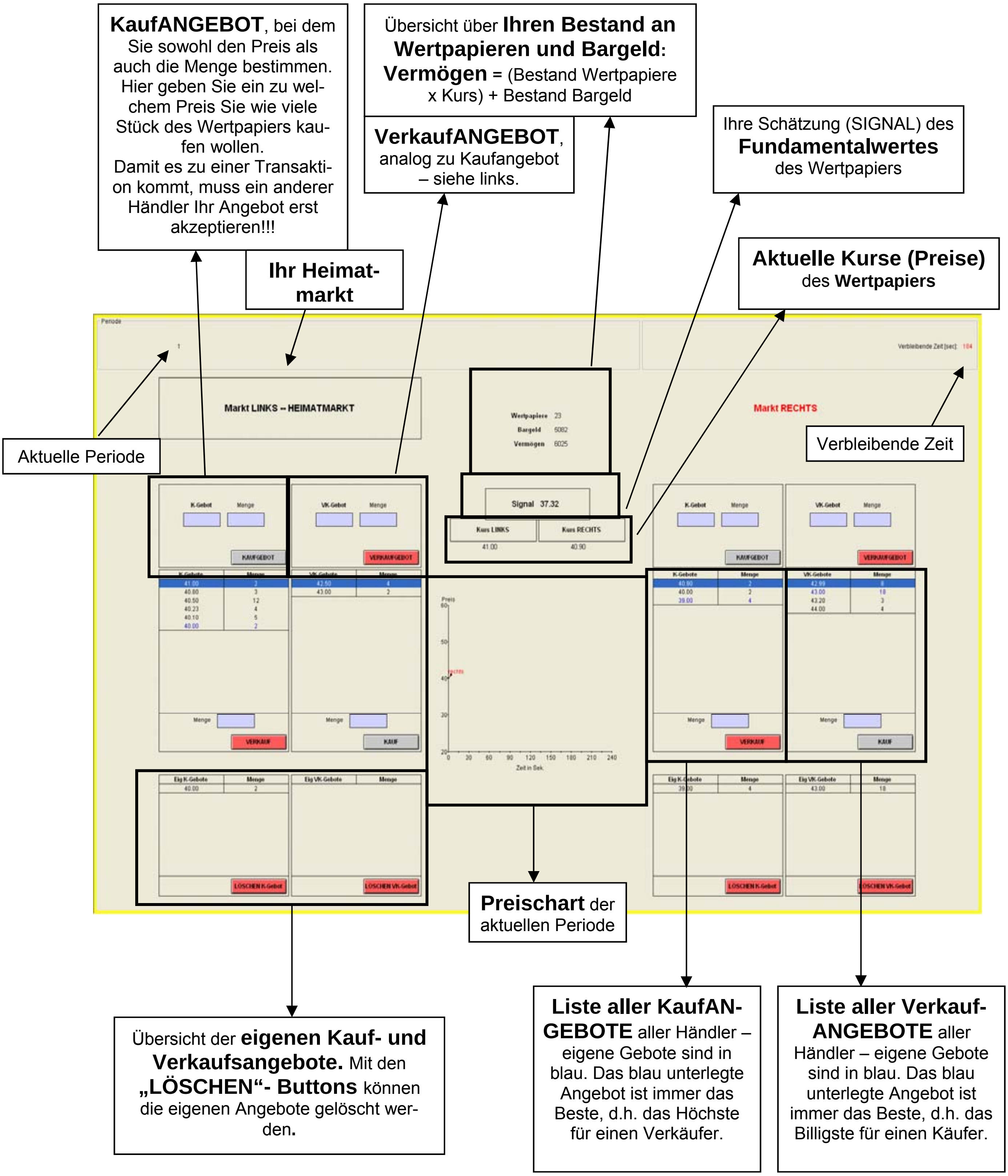

**Handel**

- Die Teilnehmer können jederzeit Wertpapiere ***kaufen*** oder ***verkaufen***, und zwar beliebig auf dem LINKEN (Ihr Heimatmarkt) und auf dem RECHTEN Markt. Zwischen den Märkten kann ohne Kosten gewechselt werden. Negative Bestände (Leerverkäufe) sind bis zu -100 an Wertpapieren und -6000 in Bargeld erlaubt. Pro Transaktion können maximal 20 Stück an Wertpapieren gehandelt werden.
- In jeder Periode steht es den Teilnehmern frei, beliebig viele ***Kauf***- und ***Verkaufgebote*** zwischen 1 und 999 abzugeben - wiederum beliebig auf dem LINKEN (Ihr Heimatmarkt) und RECHTEN Markt.
- WICHTIG: Den Kurs (Preis) des Wertpapiers bestimmen ausschließlich Sie und die restlichen 9 Händler mittels Angebot und Nachfrage.

Nach jeder Handelsperiode wird folgender History-Screen für 10 Sekunden eingeblendet:

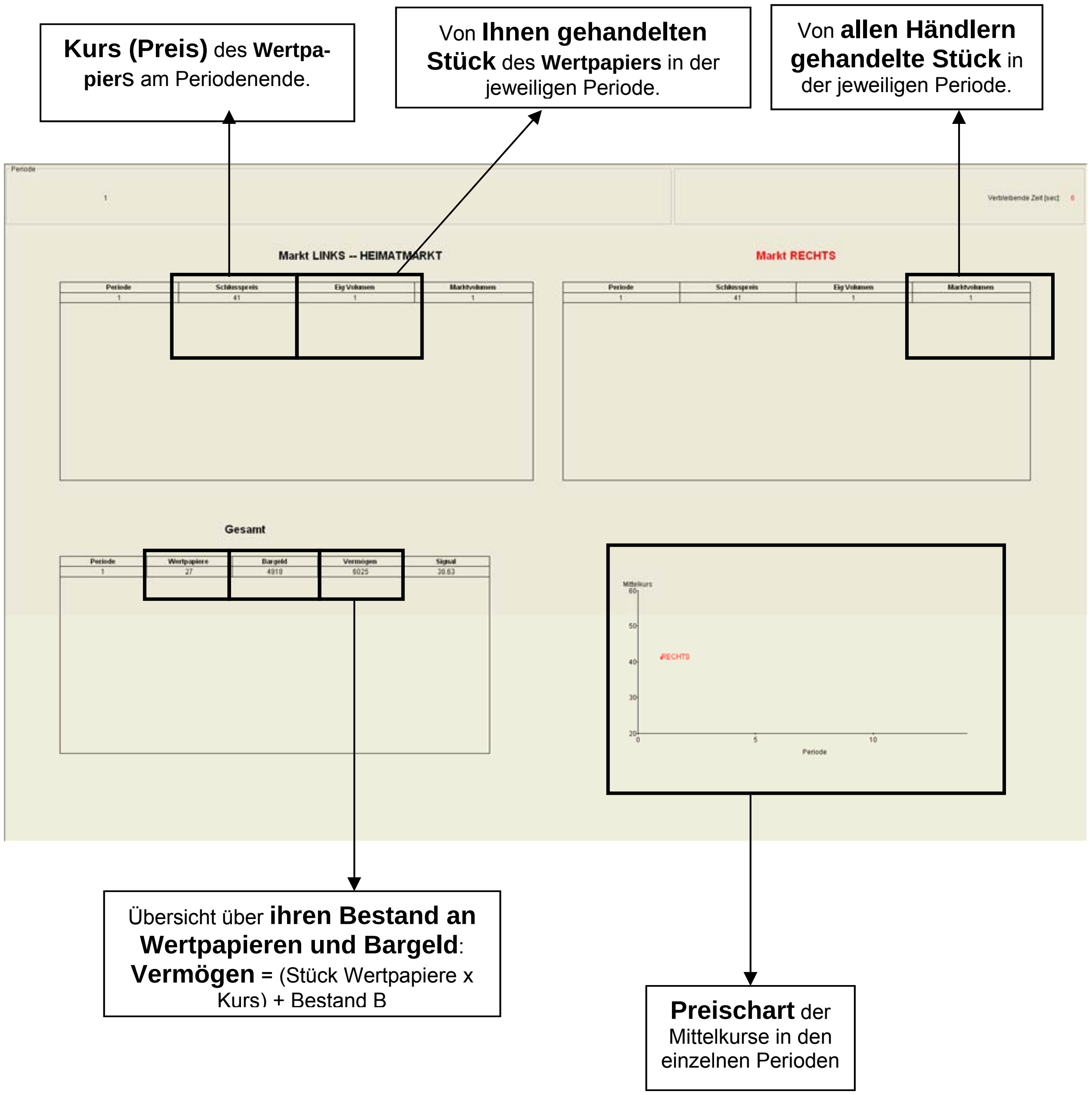

Supplement: Supplementary file 2 — Data S1. [file ECOJ-127-F610-s002.zip › EJ_MS_20140439_instruction_german.pdf]
