# Supplementary material for: Market versus Residence Principle: Experimental Evidence on the Effects of a Financial Transaction Tax
Source: Econ J (London). 2017 Oct 24;127(605):F610–31. doi: 10.1111/ecoj.12339 (PMC5698715; doi:10.1111/ecoj.12339)
Supplement: Supplementary file 2 — Data S1. [file ECOJ-127-F610-s002.zip › EJ_MS_20140439_read_me.pdf]

READ ME file "Market vs. Residence Principle: Experimental Evidence on the Effects of a Financial Transaction Tax"

This is the READ ME file to guide you through the software and data of „ Market vs. Residence Principle: Experimental Evidence on the Effects of a Financial Transaction Tax" (Manuscript Number 20140439) published in the *Economic Journal*.

Treatment Description

**Treatment**	**Description**	**#sessions**	**Market_ID**	**#Subjects**
T1 - M	Market Principle	12	1-12	120
T2 – R	Residence Principle	12	13-24	120
T3 – MR_{SAME}	Both Principles on same Market	12	25-36	120
T4 – MR_{DIFF}	Both Principles on different Markets	12	37-48	120

Software (EJ_MS_20140439_software.zip)

Six files are included. Please note that for the sake of simplicity all market software features the FV-Path A and the respective tax regime is introduced in the second half of the experiment.

- EJ_MS_20140439_software_treatment_m.ztt
- EJ_MS_20140439_software_treatment_r.ztt
- EJ_MS_20140439_software_treatment_mrsame.ztt
- EJ_MS_20140439_software_treatment_mrdiff.ztt
- EJ_MS_20140439_software_risk_aversion.ztt
- EJ_MS_20140439_software_loss_aversion.ztt

Data (EJ_MS_20140439_data.zip)

Five files are included. Stata 12 was used to analyse the data.

- EJ_MS_20140439 _data_subjects.xlsx comprises data for macro variables.
- EJ_MS_20140439 _data_fv.xlsx comprises data of the fundamental value for each market.
- EJ_MS_20140439 _data_contracts.xlsx comprises trading data for Market LEFT.
- EJ_MS_20140439 _data_contracts2.xlsx comprises trading data for Market RIGHT.
- EJ_MS_20140439 _data_questionnare.xlsx comprises data for risk and loss aversion as well as gender and age.

Instruction German (EJ_MS_20140439_instruction_german.pdf)

- Original instruction in German for home market LEFT.

EJ_MS_20140439 _data_subjects.xlsx – Variable description

treatment	1-4, indexing treatment (see description above).
markt	1-12, indexing markets within Treatments 1-4.
markt_id	1-48, indexing number of total markets.
Period	1-8, indexing period within a market.
Subject	1-10, indexing subjects within a market.
FV	
Money	Money holdings per subject in the end of each period.
Stock	Stock holdings per subject in the end of each period.
MarketOrders	Number of market orders on Market LEFT per subject in each period.
MarketOrders2	Number of market orders on Market RIGHT per subject in each period.
Contracts	Number of limit orders on Market LEFT per subject in each period.
Contracts2	Number of limit orders on Market RIGHT per subject in each period.
Volume	Trading volume on Market LEFT per subject in each period.
Volume2	Trading volume on Market RIGHT per subject in each period.
Tax	1 if Market RIGHT is taxed, 0 is default.
Tax2	1 if Market LEFT is taxed, 0 is default.

EJ_MS_20140439 _data_fv.xlsx – Variable description

treatment	1-4, indexing treatment (see description above).
markt	1-12, indexing markets within Treatments 1-4.
Period	1-8, indexing period within a market.
FV	Fundamental value for each period.

EJ_MS_20140439_data_contracts.xlsx – Variable description

treatment	1-4, indexing treatment (see description above).
markt	1-12, indexing markets within treatments 1-4.
markt_id	1-48, indexing number of total markets.
Period	1-8, indexing Period within a market.
Seller	ID 1-10, -1 is the default, -2 stands for deletion of offer by subject.
Buyer	ID 1-10, -1 is the default, -2 stands for deletion of offer by subject.
p	trading prices, 0 – 999, two decimal places, 0 is default.
q	Number of stocks traded.
qOfferB	Number of stocks posted with a limit order to buy.
qOfferS	Number of stocks posted with a limit order to sell.
contractID	1-N, indexing limit orders.
tradeID	1-N, indexing trades, 0 is default.
actionID_C	1-N, action identifier for limit orders (contracts).
actionID_T	1-N, action identifier for trades, 0 is default.
Bid	Price of open bids.
Ask	Price of open asks.
BASpreadT	Bid-Ask spread at the time of trade.
Time1	1-240, time in seconds elapsed since the beginning of a period.
Maker	TraderID of the trader who posted the limit order.

EJ_MS_20140439_data_contracts2.xlsx – Variable description

treatment	1-4, indexing treatment (see description above).
markt	1-12, indexing markets within treatments 1-4.
markt_id	1-48, indexing number of total markets.
Period	1-8, indexing Period within a market.
Seller	ID 1-10, -1 is the default, -2 stands for deletion of offer by subject.
Buyer	ID 1-10, -1 is the default, -2 stands for deletion of offer by subject.
p	trading prices, 0 – 999, two decimal places, 0 is default.
q	Number of stocks traded.
qOfferB	Number of stocks posted with a limit order to buy.
qOfferS	Number of stocks posted with a limit order to sell.
contractID	1-N, indexing limit orders.
tradeID	1-N, indexing trades, 0 is default.
actionID_C	1-N, action identifier for limit orders (contracts).
actionID_T	1-N, action identifier for trades, 0 is default.
Bid	Price of open bids.
Ask	Price of open asks.
BASpreadT	Bid-Ask spread at the time of trade.
Time1	1-240, time in seconds elapsed since the beginning of a period.
Maker	TraderID of the trader who posted the limit order.

EJ_MS_20140439_data_questionnaire.xlsx – Variable description

treatment	1-4, indexing treatment (see description above).
markt_id	1-48, indexing number of total markets.
Subject	1-10, indexing subjects within a market.
Risk	Amount invested in the risky lottery.
A1-A5	Decision of participation in each of the five lotteries per subject. 1 stands for participation, 0 stands for rejection.
Gender	1 if female, 0 otherwise.
Age	Age of experimental subject.
